# Knowledge-exchange in the Pacific: outcomes of the TROPIC (translational research for obesity prevention in communities) project

**DOI:** 10.1186/s12889-017-4254-3

**Published:** 2017-04-26

**Authors:** Peter Kremer, Helen Mavoa, Gade Waqa, Marjory Moodie, Marita McCabe, Boyd Swinburn

**Affiliations:** 1 School of Exercise and Nutrition Sciences, Deakin University, Geelong, 3216 Australia; 2 WHO Collaborating Centre for Obesity Prevention, Deakin University, Geelong, 3216 Australia; 3 College of Medicine Nursing and Health Sciences, Fiji National University, Suva, Fiji; 4 Deakin Health Economics, Faculty of Health, Deakin University, Geelong, 3216 Australia; 5 Institute for Health and Ageing, Australian Catholic University, Melbourne, 3000 Australia; 6 School of Population Health, University of Auckland, Auckland, 1142 New Zealand

**Keywords:** Obesity, Knowledge-broking, Evidence-informed policy making, Fiji

## Abstract

**Background:**

The Pacific TROPIC (Translational Research for Obesity Prevention in Communities) project aimed to design, implement and evaluate a knowledge-broking approach to evidence-informed policy making to address obesity in Fiji. This paper reports on the quantitative evaluation of the knowledge-broking intervention through assessment of participants’ perceptions of evidence use and development of policy/advocacy briefs.

**Methods:**

Selected staff from six organizations - four government Ministries and two nongovernment organizations (NGOs) - participated in the project. The intervention comprised workshops and supported development of policy/advocacy briefs. Workshops addressed obesity and policy cycles and developing participants’ skills in accessing, assessing, adapting and applying relevant evidence. A knowledge-broking team supported participants individually and/or in small groups to develop evidence-informed policy/advocacy briefs. A questionnaire survey that included workplace and demographic items and the self-assessment tool “Is Research Working for You?” (IRWFY) was administered pre- and post-intervention.

**Results:**

Forty nine individuals (55% female, 69% 21–40 years, 69% middle-senior managers) participated in the study. The duration and level of participant engagement with the intervention activities varied – just over half participated for 10+ months, just under half attended most workshops and approximately one third produced one or more policy briefs. There were few reliable changes on the IRWFY scales following the intervention; while positive changes were found on several scales, these effects were small (*d* < .2) and only one individual scale (assess) was statistically significant (*p* < .05). Follow up (*N* = 1) analyses of individual-level change indicated that while 63% of participants reported increased research utilization post-intervention, this proportion was not different to chance levels. Similar analysis using scores aggregated by organization also revealed no organizational-level change post-intervention.

**Conclusions:**

This study empirically evaluated a knowledge-broking program that aimed to extend evidence-informed policy making skills and development of a suite of national policy briefs designed to increase the enactment of obesity-related policies. The findings failed to indicate reliable improvements in research utilization at either the individual or organizational level. Factors associated with fidelity and intervention dose as well as challenges related to organizational support and the measurement of research utilization, are discussed and recommendations for future research presented.

## Background

The global increase in obesity prevalence is a major public health concern requiring a multi-faceted systems approach [1]. An important aspect to resolving obesity is the development of a suite of evidence-informed policies to shape obesogenic attitudes and behaviors [2]. Integrating relevant research evidence into appropriate and effective public policy is challenging, given that researchers and policymakers frequently have different agendas, timelines and priorities that constrain the use of evidence to inform policies [3]. Effective exchange of knowledge between evidence-producers (researchers and others) and end-users (those who initiate, select, approve, implement and evaluate policy) is critical to evidence-based policy development [3].

Effective knowledge exchange is determined by: 1) researchers producing timely and relevant evidence; 2) policy-makers communicating their priorities and timelines; and 3) policy-makers having the skills and resources to utilize evidence to inform policies [4]. Focusing on the third of these points, effective use of evidence to inform policy is determined by the ability of policy makers to access and critically analyse the best available evidence and apply it to policy formulation. The best evidence is accessible (available, affordable, appropriately framed), relevant (to obesity and the local context) and timely [4]. Evidence-informed policy making is, however, complex and often requires a change in organizational culture to ensure that the evidence is actually identified, utilized and converted into policy. Therefore, researchers need to understand both policy making processes and timelines, as well as the culture in which policy formulation occurs in order to optimize opportunities for evidence-informed policy making [5]. Indeed, organizational components are stronger predictors of evidence-informed policy making than individual factors [3, 5, 6]. It is also important that advocacy documents are informed by relevant evidence, given the potential for advocates to influence policy [7].

Strategies that enable evidence-informed policy making include: 1) producing relevant evidence that is aligned with policy cycles; 2) extending policy developers’ evidence-informed policy making skills and utilization of evidence; 3) working with policy developers and organizations to develop individual and organizational cultures that value and support evidence-informed policy making; 4) facilitating strong relationships between researchers, policy developers and policy making organizations [8, 9] and 5) embedding evidence-informed policy making into policies and practices [4].

Expertise and resources to enable evidence-informed policy making are often limited, especially in low to middle income countries, and those with small populations. This study reports on the evaluation of the impact of a 3-year knowledge exchange project called Translational Research for Obesity Prevention in Communities (TROPIC) [4] conducted in the Republic of Fiji from July 2009 to April 2012. The specific objectives of the TROPIC project were to: 1) extend evidence-informed decision making skills in selected partner organizations; 2) use a knowledge-broking approach to increase the uptake of evidence from the Obesity Prevention in Communities (OPIC) project [10, 11] and other relevant sources in the development of obesity-related policy and embed this in policy and advocacy documents; and 3) facilitate changes in organizational culture so that organizational structures were supportive of evidence-informed policy making. Results of the qualitative evaluation of the TROPIC project have been reported previously [12, 13]. Consequently, this paper reports on the quantitative evaluation of the TROPIC project and specifically on changes in self-reported evidence-informed policy making skills developed among nominated employees in selected government ministries and nongovernment organizations (NGOs).

## Methods

### Design

The TROPIC project incorporated a pre-post intervention design. Specific details of the project have been reported previously [4, 12, 13].

### Organizations and participants

We elected to recruit a maximum of six organizations in order for the small TROPIC knowledge-broking team to have sufficient resources to facilitate evidence-informed policy making skills for development of policy briefs. Eight government and two NGOs were identified as potential participant-organizations based on: their potential to make or influence policies that improve food and/or physical activity environments; wide demographic (ethnic group; religion; urban/rural) representation to ensure a broad reach; capacity to release and support staff to participate in TROPIC activities; potential to share evidence-informed policy making knowledge and skills within their own and other organizations; and previous relationships with the research team [4, 5, 14]. High-level meetings subsequently took place with either government ministers or permanent secretaries (deputy ministers) in government organizations, or chief executive officers from NGOs [13]. Two of the six government organizations approached declined the invitation to participate in the study citing lack of organizational resources (time, staff) and difficulty aligning the project to their policy cycles. Both of the NGOs that were approached agreed to participate [13]. Each participating organization nominated a senior staff member as a focal/contact person. In addition, each organization nominated between 5 and 12 staff members who were either currently engaged in policy development, or were likely to do so in the near future, to participate in the study.

### Intervention

In brief, the intervention involved a knowledge-broking team delivering a 12–18 month program per organization. The program comprised workshops that targeted evidence-informed policy making skills and practical support for developing evidence-informed policy briefs and advocacy statements to reduce obesity (we subsequently use the term ‘policy briefs’ to represent both of these) [4, 13]. The broad framework of the intervention was the same across organizations, but the specific content of the program was tailored to each of the six participating organizations. Knowledge-broking strategies included identifying policy/advocacy topics that could potentially reduce obesogenic environments, monitoring, evaluating and facilitating time-management skills, accommodating other organizational and individual priorities, delivering workshops, conducting meetings (whole group, small group, one-to-one) and supporting individual writing of policy briefs [4]. A number of these strategies were tailored by organization. For example, the policy/advocacy topics were negotiated so that they were aligned to the plans of individual organizations [4, 13]. The number and focus of the workshops were also tailored according to the needs of the organizations [4]. For example, additional workshops that provided an introduction to non-communicable diseases (NCDs) as well as the burden of NCDs and social and economic impacts, were provided to non-health organizations. Similarly, there was variable understanding of what constituted a policy and the local policy cycle across organizations meaning that the focus of the workshop activities were varied. Policy brief templates were also tailored for each organization - while many components of these templates were consistent, other components were specifically tailored according to organizational requirements [13]. One organization (with an existing policy unit) had an existing internal policy brief template and was hesitant to use the TROPIC template. The internal policy brief template, however, had some deficiencies (eg. no provision for evidence to support/justify the policy issue) and after negotiation, it was resolved that the policy brief would be drafted using both templates.

### Project measures

The intervention’s effectiveness in increasing the use of evidence in the development of policy briefs to reduce obesity was assessed through four forms of data. First, individual participants’ perceptions of their knowledge and experience with evidence and policy were elicited via semi-structured interviews prior to engagement in the study [13]. Second, a three part survey questionnaire was used to capture information pre and post the TROPIC program to enable quantitative assessment of any changes in the valuing and utilization of evidence-informed decision making at both individual and organizational levels. Third, individual and organizational perceptions of the TROPIC program were elicited via individual interviews conducted with two groups at the conclusion of the program: 1) all participants in the TROPIC program; and 2) a high-level officer from each participating organization. Fourth, the knowledge-broking team recorded individual and organizational processes in terms of time and resources for workshop participation and development of policy briefs. They also documented outputs arising from TROPIC, specifically the completion of policy briefs, as well as evidence of the embedding of evidence-informed decision making practice. Given that results obtained from both sets of interviews and the diary data have been reported previously [12, 13], this paper focuses on the quantitative survey data.

### Survey instrument

A three part survey questionnaire was used to collect information. The first two parts of the survey utilized the “*Is Research Working for You? A Self-Assessment Tool and Discussion Guide for Health Services Management and Policy Organizations*” (IRWFY). In consultation with a range of experienced researchers, we selected the IRWFY tool as one of the few available at project set-up that could provide a quantitative measure of both individual and organizational research utilization. The IRWFY self-assessment tool was developed by the Canadian Health Services Research Foundation [15] to assist health service delivery organizations to examine organizational strengths and weaknesses in evidence-informed decision making. The IRWFY tool measures perceptions of organizational culture (values; attitudes to evidence use; intentions to use evidence), and use of evidence (access; adapt; use in decision making; adopt) at both organizational (part one of survey) and individual (part two of survey) levels. Part one quantifies individual perceptions of how a participant’s organization 1) acquires and 2) uses research. Each of these two components is measured via a number of separate scales. The *acquire* component includes a single “acquire” scale (19 items and 1 open response item) made up of two subscales - an “ability/resources” subscale (10 items) and a “looking for research” subscale (9 items). The *uses research* component includes four scales: “assess” (6 items and 1 open response item), “adapt” (11 items and 1 open response item), “apply” (11 items and 1 open response item), and “decision making processes” (6 items and 1 open response item). Part two quantifies individual’s perceptions of their own *ability to use, understand and transfer research evidence*. This component is measured via three scales: “looking for research” (9 items), “assess” (6 items), and “adapt” (8 items and 1 open response item). A 7-point response scale (1 = ‘strongly agree’ - 5 = ‘strongly disagree’, 6 = ‘don’t know’, 7 = ‘not applicable’) is used for each of the scale items. The IRWFY tool has demonstrated good usability, strong response variability and adequate discriminant validity [16]. The third part of the survey was used to capture additional information about the participant’s perceptions of challenges to utilization of research evidence in their organization, strengths of their organization in the use of research evidence for decision making, and any other comments about the use of research evidence in their organization (three separate open response items) as well as information about participants’ past and current employment in their organization/sector, training/education on how to do research, and a question where they rated their own understanding of research (5-point response scale: 1 = ‘poor’; 5 = ‘excellent’) as well as general demographic information.

### Consent and ethics approval

The TROPIC project was a three-year study, funded by the Australian Agency for International Development (AusAID) on an Australian Development Research Awards (ADRA0800148) grant. The project was approved by the Deakin University Human Research Ethics Committee (2009-142), the Fiji Health Research Committee, and the Fiji National Research Ethics Review Committee (2009-308). All participants provided informed written consent prior to engaging in the study.

### Data treatment and analysis

Descriptive statistics (proportions) were used to summarize demographic and relevant background information. Participant’s level of engagement with the intervention was also summarized descriptively according to three separate measures – the duration of participation, workshop attendance, and policy/advocacy brief completion. The duration of participation was determined as the period from when the participant entered the study (by completing the consent requirement) through to either: 1) completion of their final policy brief, 2) the point where the participant ceased their involvement in the study, or 3) the termination of the overall project. Workshop attendance was calculated as the proportion of workshops attended by each participant relative to the number offered/available to the participant. Policy brief completion was determined from the number of policy briefs fully completed by individual participants - incomplete briefs were not included.

All IRWFY items were checked for missing and out of range values. Items were reverse scored to aid interpretability (ie. higher scores representing ‘better’ research utilization) and ‘don’t know’ and ‘not applicable’ responses were specified as ‘missing’. Organizational and individual scale scores (means) were computed using responses from the relevant items. Pro-rated means were computed where scales included items with missing data, providing data were available for most (>70%) items. An overall ‘mean organizational’ and ‘mean individual’ score was also computed using all valid scores from items from the five organizational scales and three individual scales respectively and these scores represented a global score on ‘research utilization’. A variable representing the overall level of participant engagement with the TROPIC project was generated by summing dichotomized versions of the workshop attendance (0 = <50% attendance; 1 = ≥ 50% attendance) and policy brief completion (0 = none; 1 = one or more) measures. Low engagement was indicated by a summed score of 0, moderate engagement a score of 1 and high engagement a score of 2. Scale reliability (internal consistency) was assessed using Cronbach’s alpha and evaluated using the following guidelines: >.9 ‘excellent’, >.8 ‘good’, >.7 ‘acceptable’, >.6 ‘questionable’, >.5 ‘poor’, and <.5 ‘unacceptable’ [17]. Changes in pre- and post-intervention scores on the IRWFY organizational and individual scales and the self-rating understanding of research were assessed. At the individual level, responses from all individuals with pre- and post-intervention data were used to compute individual-level means and differences were tested using separate t-tests. Due to small numbers in ‘engagement level’ subgroups, descriptive statistics (means, standard deviations) were used to examine whether participants’ level of project engagement influenced pre- and post-intervention differences on the five organizational and three individual scales. Due to the limitations of sample size, intervention dose and sensitivity of the IRWFY instrument, we also assessed pre- and post-intervention change - at both the individual- (ie. a series of N of 1 ‘studies’) and organizational-levels (ie. case studies for six organizations) – by comparing the overall mean individual and mean organizational values at post-intervention with the corresponding pre-intervention values. To generate the mean overall organization level score, we aggregated the individual responses by organization and divided this value by the number of staff from the organization providing data at both time-points. Individual- and organizational-level change scores were computed by subtracting the pre-intervention score from the post-intervention score and these change scores were then plotted. A positive change score indicated an increase in research utilization, a negative change score indicated a decrease in research utilization, and a zero score no change. Visual analysis and tests for proportional change (*Z* statistic) were used to interpret the plotted change scores. All data entry, preparation and analysis was performed using SPSS V22 (IBM Inc.) and statistical significance was accepted as *p* < .05 for all effects.

## Results

### TROPIC sample

A total of 49 individuals participated in the study. Characteristics of the sample participants at commencement are summarized in Table 1. In general, there was a relatively even spread of participants aged between 21 and 50 years and a slightly higher proportion of females. Almost half had worked in the ‘policy/advocacy sector’ for 12 or more years, about one third had been employed with their organization for 12 years of more, most held middle or senior management positions and had been in their current position for less than 3 years. Most participants reported having a ‘good’ to ‘very good’ understanding of research, and over half of the sample reported receiving research training/education through undergraduate classes and/or on-the-job training/collaboration. One participant reported no previous research training/education.

**Table 1:** Characteristics of TROPIC participants at pre-intervention (*n* = 49)

Variable	*n* (%)
Gender	
Male	22 (44.9)
Female	27 (55.1)
Age group	
21–30 yrs	17 (34.7)
31–40 yrs	17 (34.7)
41–50 yrs	15 (30.6)
> 50 yrs	0 (0)
Highest completed or current education	
Professional qualification	13 (26.5)
Bachelors degree	15 (30.6)
Postgraduate diploma	14 (28.6)
Masters degree	7 (14.3)
Organization	
Organization 1	12 (24.5)
Organization 2	12 (24.5)
Organization 3	5 (10.2)
Organization 4	9 (18.4)
Organization 5	7 (14.3)
Organization 6	4 (8.2)
Position^a^	
Board member	0 (0)
Senior manager	8 (16.3)
Middle manager	26 (53.1)
Senior clinician	0 (0)
Front line staff	6 (12.2)
Other (eg. technical staff, program assistant)	5 (10.2)
Years in current position^a^	
< 1 yr	17 (37.8)
1 yr – <3 yrs	12 (26.7)
3 yrs – < 6 yrs	12 (26.7)
6 yrs – < 11 yrs	1 (2.2)
≥ 12 yrs	3 (6.6)
Years with current organization^a^	
< 1 yr	8 (17.8)
1 yr – <3 yrs	7 (15.6)
3 yrs – < 6 yrs	8 (17.8)
6 yrs – < 11 yrs	3 (6.7)
≥ 12 yrs	19 (42.2)
Years worked in sector^a^	
< 1 yr	5 (11.1)
1 yr – <3 yrs	6 (13.3)
3 yrs – < 6 yrs	7 (15.6)
6 yrs – < 11 yrs	5 (11.1)
≥ 12 yrs	22 (48.9)
Research training/education^a^	
None at all (yes)	1 (2.2)
Via undergraduate classes (yes)	26 (57.8)
Via postgraduate classes (yes)	9 (18.4)
Via Masters/PhD studies (yes)	14 (31.1)
Via clinical work (yes)	2 (4.4)
Via on-the-job collaboration (yes)	23 (51.1)
Via part of research team (yes)	16 (35.6)
Via other (yes)	4 (8.9)
Understanding of research^a^	
Excellent	4 (8.9)
Very good	12 (26.7)
Good	26 (57.8)
Fair	3 (6.7)
Poor	0 (0)

^a^No survey data for *n* = 4 participants

### Engagement in TROPIC intervention

The profile of participant engagement is summarized in Table 2. Whilst almost half of the participants were engaged over the full duration of their organization’s engagement in the project, approximately one third were engaged for 3 months or less. Just under half of the participants attended 75% or more of the workshops available to them, and similarly, just under half attended less than 50% of the available workshops. Only a small proportion (8%) of participants completed more than one policy brief and about two thirds failed to complete a single brief. One organization elected for its participants to develop a single policy brief as a joint exercise. The pattern of engagement was generally similar across the six organizations, although there were some differences. For example, higher proportions of staff from Organization 3 (80%) and Organization 1 (75%) participated for the full project duration relative to the other organizations. Relative to staff from the other organizations, a higher proportion of staff from Organization 1 attended most of the available workshops (66%) and developed at least one policy brief (58%) while a higher proportion of staff from Organization 2 attended fewest of the available workshops (75%) and developed no policy briefs (83%).

**Table 2:** Summary of individual- and organization-level of engagement with the TROPIC intervention

	All (*n* = 49)	Org1 (*n* = 12)	Org2 (*n* = 12)	Org3 (*n* = 5)	Org4 (*n* = 9)	Org5 (*n* = 7)	Org6 (*n* = 4)
Duration of engagement							
< 1 mth	9	1	3	1	1	2	1
1–3 mths	7	2	2	0	1	0	2
4–9 mths	8	0	3	0	5	0	0
10–17 mths	25	9	4	4	2	5	1
Workshop attendance^a^							
Less than 25%	7	4	5	0	0	2	0
25% < 50%	15	0	4	1	3	0	3
50% < 75%	4	0	0	2	1	1	0
75% or more	23	8	3	2	5	4	1
Policy briefs developed							
0 briefs	32	5	10	2	7	5	3
1 brief	13	5	0	3	2	2	1
2 briefs	4	2	2	0	0	0	0

^a^Calculated as number offered/number attended

### IRWFY instrument

Internal reliability coefficients for the five organizational and three individual scales of the IRWFY instrument, at pre- and post-intervention, were computed. Results are shown in Table 3 and indicate that the IRWFY scales had acceptable – excellent internal reliability [17].

**Table 3:** Scale statistics for IRWFY instrument pre- and post-intervention

		Pre-intervention		Post-intervention	
Scale	Number of items	Total scale^a^ (*M, SD*)	α	Total scale^a^ (*M, SD*)	*α*
Organizational					
Acquire	19	69.8 (10.9)	.88	62.9 (8.7)	.82
Assess	6	21.0 (4.4)	.86	20.0 (4.9)	.90
Adapt	11	36.8 (7.6)	.90	36.8 (7.3)	.89
Apply	11	38.8 (8.3)	.92	37.0 (6.9)	.91
Decision making	6	20.8 (5.3)	.92	20.1 (5.3)	.92
Individual					
Looking for research	9	33.1 (5.8)	.85	32.3 (5.3)	.82
Assess	6	21.0 (4.3)	.88	22.1 (3.0)	.75
Adapt	8	28.7 (6.0)	.91	27.7 (5.5)	.92

^a^Derived from summing responses on items scaled 1 = ‘strongly disagree’, 5 = ‘strongly agree’

### Intervention effect

Participants’ perceptions of how well they use research and how well their organization uses research before and after the TROPIC intervention were indicated by scores on the IRWFY scales. Means for each of these scales are summarized in Table 4. There were few differences between pre- and post-intervention means across the five organizational scales. Furthermore, t-tests revealed no statistically significant differences (ie. pre-post intervention change) and effect sizes for each of the scales were small. It should be reiterated here that the analyzed scores are *individuals’ perceptions* of their organization and not the means of a collection of individuals from the same organization. Unfortunately, the small within-organization subsample numbers precluded this level of analyses (however, in the following section, we do present a summary of an overall score that is aggregated by organization). The means for the three individual scales all show an increase in research utilization at post-intervention. However, only the effect for the “assess” scale was statistically significant and effect sizes were in the small to moderate range. There was also no difference for the individual self-rating of understanding of research from pre- (*M* = 2.7, *SD* = 0.8) to post-intervention (*M* = 2.7, *SD* = 0.8) (*t* = .00, *p* = 1.00, *d* = .00).

**Table 4:** Descriptive (mean, standard deviation) and test statistics for IRWFY scales at pre- and post-intervention (*n* = 32)

		Pre-intervention		Post-intervention				
Scale	*n*	*M* ^a^	*SD*	*M* ^a^	*SD*	*t*	*p*	*d*
Organizational								
Acquire	32	3.3	0.6	3.3	0.5	.83	.41	−.04
Assess	32	3.4	0.7	3.3	0.8	.68	.50	.13
Adapt	32	3.2	0.7	3.4	0.6	−.96	.35	−.17
Apply	32	3.4	0.8	3.3	0.7	.90	.38	.15
Decision making	32	3.4	0.7	3.4	0.9	−.02	.99	.00
Individual								
Looking for research	32	3.6	0.7	3.6	0.6	−.38	.71	−.06
Assess	31	3.4	0.7	3.7	0.5	−2.37	.03	−.43
Adapt	30	3.5	0.7	3.6	0.6	−1.02	.32	−.19

^a^Response scale: 1 = ‘strongly disagree’, 5 = ‘strongly agree’

Since the intervention dose (ie. individual engagement with the intervention activities) varied between individuals, we also examined the effect of level of dose on scores on the individual and organizational scales. Pre- and post-intervention means were computed for each IRWFY scale according to level of individual engagement – indicated by an aggregate measure captured by the workshop attendance and policy brief development variables (see Table 5). The small group numbers precluded formal statistical testing. Nevertheless, descriptive assessment of the pre- and post-intervention scores on the five organizational scales failed to indicate any possible effect of engagement. Similarly, there were few differences on the three individual scales, although the improvements for the moderate engagement group, and to a lesser extent high engagement group, were generally greater than those observed for the low engagement group.

**Table 5:** Descriptives statistics (mean, standard deviation) for IRWFY scales pre- and post-intervention by summary measure of engagement

	Low^a^		Moderate		High	
Scale	Pre (*n* = 8)	Post (*n* = 8)	Pre (*n* = 8)	Post (*n* = 8)	Pre (*n* = 16)	Post (*n* = 16)
Organizational						
Acquire	3.3 (0.6)	3.3 (0.5)	3.3 (0.7)	3.4 (0.5)	3.4 (0.4)	3.2 (0.4)
Assess	3.6 (0.5)	3.5 (0.8)	3.2 (0.9)	3.3 (0.7)	3.4 (0.7)	3.3 (0.9)
Adapt	3.2 (0.9)	3.4 (0.5)	3.2 (0.7)	3.4 (0.8)	3.3 (0.6)	3.3 (0.6)
Apply	3.4 (0.8)	3.3 (0.6)	3.5 (1.0)	3.5 (0.6)	3.4 (0.7)	3.2 (0.7)
Decision making	3.5 (0.9)	3.5 (1.1)	3.4 (0.9)	3.6 (1.0)	3.3 (0.5)	3.2 (0.7)
Individual						
Looking for research	3.5 (0.8)	3.5 (0.7)	3.6 (0.8)	3.8 (0.8)	3.6 (0.6)	3.5 (0.5)
Assess	3.5 (0.4)	3.4 (0.6)	3.2 (0.7)	3.9 (0.5)	3.5 (0.8)	3.8 (0.5)
Adapt	3.5 (0.7)	3.4 (0.7)	3.4 (0.8)	3.8 (0.4)	3.5 (0.7)	3.5 (0.7)

^a^Measure of engagement computed by summing proportion of workshop attendance (0 < 50%; 1 ≥ 50%) and policy brief completion (0 = none; 1 = at least one completed); low engagement: summed score of 0, moderate engagement: summed score of 1, high engagement: summed score of 2

**Fig. 1:**
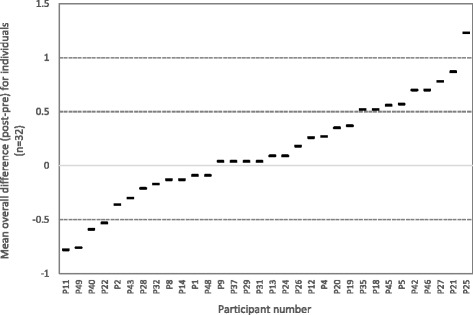
Mean overall difference (post-intervention – pre-intervention) across all individual items for individual participants

**Fig. 2:**
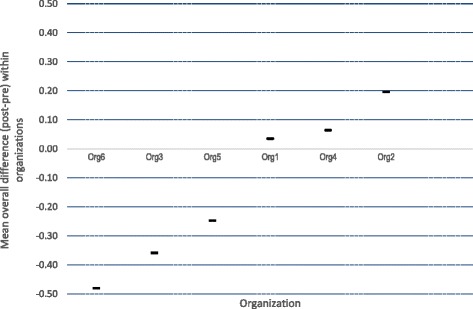
Mean overall difference (post-intervention – pre-intervention) across all organizational items for participants aggregated by organization

Since grouped analysis of the data may conceal small but potentially important changes, we also examined differences across pre- and post-intervention by computing both individual and organizational (ie. aggregated scores of individuals from the same organization) change scores as shown in Figs. 1 and 2 respectively. Of the individual participants having both pre- and post-intervention scores, 63% indicated increased research utilization post-intervention (37% indicated decreased research utilization). This effect, however, was not significantly different from chance level proportions (*Z* = −1.01, *p* = .31). Of the six organizations, the aggregated individual staff responses indicated that at an organizational level, 50% of the participating organizations had increased their research utilization post-intervention whilst 50% had decreased their research utilization.

## Discussion

This paper reported the quantitative results of the effect of a knowledge-broking team on the use of obesity-related evidence in policy briefs. Specifically, it reported on changes in perceptions of evidence-informed policy making skills among employees in four selected government ministries and two NGOs. The findings indicated no changes in participants’ perceptions of their organization’s utilization of research, however modest positive changes (increases) were observed on the three individual-level scales although only one effect (“assess” scale) was significant. Findings from follow up descriptive analyses failed to reveal any ‘dose’ effect – there was no consistent evidence indicating greater (positive) change for those participants who more strongly engaged with the TROPIC program although changes were marginally stronger for those categorized as moderately or highly engaged compared with those lowly engaged. There was also no effect on individual’s global self-rating of understanding of research. Findings from analyses of perceptions at the (aggregated) organizational level and individual level indicated that effects (ie. changes in perceptions in research utilization) were not different to those expected by chance.

Overall, the quantitative findings reported herein, generally failed to show any consistent effects for the TROPIC knowledge exchange research program. The only significant effect was for the individual scale of “assess” where scores were improved - indicating that individual participants perceived that their ability to critically evaluate research methodology, assess the relevance of research and synthesize different pieces of research, was better after the TROPIC intervention. These specific results are consistent with previously reported findings from the interview data [12]. While the results for the two other individual scales (ie. “looking for research”, “adapt”) showed positive changes, these effects were smaller in magnitude and non-significant. The interview findings provide some explanation for the modest effects; participants indicated that accessing research was limited by resources and infrastructure (access to databases, computers, internet), as well as time constraints and in some cases, a perceived lack of organizational support [12]. The failure of the TROPIC intervention to show any significant effects at the organizational-level is not surprising since it is unlikely that adjusting the capabilities and skills of a small number of personnel within any one organization would be sufficient to alter the perceptions of research utilization capability at an organizational level. Even when individual data was aggregated by organization, no positive effects were discerned. Thus, while there was some indication of positive change in research utilization at an individual level, there was no evidence of perceived change at the organizational level suggesting that the reach of the TROPIC intervention within the participating organizations was limited. It is well recognized that shifts in organizational culture require multi-faceted approaches over time [9, 18]. Exploration of the factors that might facilitate or impede the reach of similar interventions need to be clarified.

The application of TROPIC knowledge exchange research program through a knowledge-broking approach is important given the explanatory nature of the culture and behavioral factors that influence decision making. While Waqa, Mavoa et al. [12] have previously reported that many participants believed they had increased skills in acquiring, accessing, adapting and applying evidence following the TROPIC project, and that their reporting had become based more on credible research evidence rather than perceptions and anecdotal “evidence”, the results of the quantitative evaluation failed to reveal consistent intervention effects at an individual level and no effect at an organizational level. A number of factors may account for this apparent inconsistency of effects. First, while Waqa, Mavoa et al. [12] captured qualitative impressions of the TROPIC intervention, the present study reports only on quantitative outcomes. Given the myriad of challenges faced when operationalizing the intervention, discerning (possible) intervention effects through quantitative assessment is problematic and unlikely to be able to capture the more personalized perceptions of the benefits of the intervention. Second, a number of participants commented in their post-intervention interviews that they had overrated their knowledge of research when they entered the TROPIC program and completed the initial IRWFY survey. It was only when they gained an increased understanding of research and its application that they felt they were able to provide a realistic score. Third, high staff turn-over in all six organizations compromised continuity of staff engagement in the program. Fourth, many participants had roles that required multiple tasks, resulting in fragmented and limited time to allocate to TROPIC activities. Natural disasters (ie. a cyclone and two major floods) and concomitant public health emergencies further fragmented time available to engage in TROPIC; Fiji experienced several natural disasters that necessitated governmental and NGO action which subsequently diverted staff from attending workshops and completing policy briefs. Fifth, only about one third of the organizations received tangible high-level political support from the relevant ministers (indicated by the presence of permanent secretaries (deputy ministers) and directors during presentation of policy briefs to the organization). The importance of high-level organizational support was reflected in the higher number of policy briefs produced by these organizations.

A number of other organizational barriers also limited the impact of using evidence in the development of policy briefs. Whilst these barriers varied across the six participating organizations, they included factors such as the reallocation of participants onto other activities despite their reported interest in the intervention activities and broader project, lack of organizational support and incentives to persist with policy development work in the face of other organizational priorities as well as the lack of information and technology resources (eg. database software) to enable storage of extracted evidence from scientific literature. Limitations of this nature have been reported elsewhere [19] and remain a challenge to embedding evidence-informed policy making into organizations. These challenges are heightened in low to middle income countries with limited economic and human resources and less capacity to either access or adapt evidence for policy documents [20], or foster a culture that supports and extends evidence-informed policy making [4, 21]. These limitations make it difficult to foster and sustain a culture, structures and processes that support evidence-informed policy making within organizations.

More generally, the relatively short duration of TROPIC program (12–18 months per organization) may have been insufficient for the program activities to be integrated into the individual participants’ working practices but more especially the practices of the organization within which the participants operated. Almost half of the participants had less than 9 months engaged in the TROPIC program. It has been suggested that 15–18 months is the shortest duration that can be expected to produce change in evidence-informed decision making [8, 22], however, competing priorities of participating organizations, the timing of entry into the project vis-a-vis organizational policy cycles, and limited resources of the TROPIC team meant that it was not possible to provide each organization with the optimal duration of intervention. The effectiveness of future knowledge-broking programs may require a longer duration, as well as greater staff engagement – that is, both a higher proportion of staff within organizations need to be engaged and staff also need to be provided with support to continue their participation in all of the intervention activities in the face of other more important priorities. Consideration of these and other factors, including longer training periods and more individualized tailoring of intervention strategies, as well as evaluation designs that allow sufficient time for effects to permeate through the culture of organizations before post-intervention assessment, is needed before measurable shifts in the utilization of research can be discerned.

Another factor that may have contributed to the limited (quantitative) effects is our choice of survey instrument. We used the IRWFY instrument, one of the few available in 2009. The instrument has good usability, strong response variability and adequate discriminant validity [16], however it was not designed to assess intervention effects but rather for organizational self-assessment, to ‘scan’ and generate discussion about how research is used. Thus, it is possible that the IRWFY tool was not sufficiently sensitive to enable detection of relatively small changes in individual or organizational knowledge and practices. This, in conjunction with the relatively small final sample at post-intervention may further explain the limited and inconsistent findings. Nevertheless, the magnitude of the observed effects (small) indicate that even though the potential for finding a “statistically significant” intervention effect may have been low, the observed effects were small in any case and point to other more program-specific factors as we have noted.

While multiple competing priorities in most participating organizations limited the impact of using evidence in policy briefs, the knowledge-broking team offered a flexible schedule of activities, organized the workshops away from participants’ workplace and in a block as well as providing a sequence of policy brief writing retreats that were timed to be accessible for participants in each organization. In the interviews, participants indicated that barriers to evidence-informed policy making were not just individual lack of knowledge about data sources, but also organizational. Participants cited inadequate time to develop evidence-informed briefs, and insufficient resources for accessing and managing evidence as barriers [12]. Embedding of evidence-informed policy making within organizational structures requires a critical mass of people with skills to acquire, assess and adapt evidence to inform policy, the availability of timely, relevant evidence in language that resonates for policy-makers, an organizational culture where there are clear structures and processes in place to support evidence-informed decision making, and that recognises and rewards the use of evidence-informed decision making, and strong researcher-end-user relationships [21]. Organizations who participated in TROPIC are now well placed to build on: 1) excellent relationships with researchers, and 2) the growing number of personnel who have acquired evidence-informed decision making skills. The next challenge is to continue to develop a culture that builds a solid organizational infrastructure to support evidence-informed decision making that informs all policies that have potential health benefits. Post-TROPIC initiatives to seek a similar experience for more personnel by at least one of the participating organizations suggests that there is motivation to continue to build a critical mass of staff with evidence-informed policy making skills.

The TROPIC study has provided some insights of knowledge-broking approaches to support evidence-informed policy development that are generic and can be transferred to any policy area. The value placed on types of evidence within decision making contexts is dependent on individuals, the organizations in which they work and the systems in which they operate. Decision making processes are also context-dependent [23]. A supportive organizational environment is especially important in the transferability of skills in any low- or middle-income country with limited policy making resources. This observation is consistent with other studies [4, 20, 21].

Strengths of this study include its uniqueness in a number of respects. The knowledge-broking team employed a number of complementary approaches to facilitate evidence-informed policy making, including specific workshops tailored to the needs of individual organizations as well as individual knowledge-broking sessions whereby participants received personalized guidance about accessing and utilizing research evidence to inform development of policy briefs. The knowledge-broking team also provided broader mentoring support for individual participants, assisted in aligning timelines for policy brief completion to policy timelines such as approval of annual plans, protecting time and also sharpening their understanding of how to plan and draft policy briefs. The team also developed a policy brief template that guided writing, as well as a template for presenting briefs to higher decision making levels [13]. A number of limitations of the study are acknowledged. Participants’ lack of understanding of what constitutes evidence resulted in many participants overestimating their evidence-informed policy making skills when entering the intervention [13]. This overestimation, along with the disparity in participants’ basic skills and availability required more individual mentoring than the team had anticipated. The project timelines and capacity limits of the TROPIC team, meant that it was not possible to individually tailor each of the knowledge-broking strategies. The duration of the intervention (≤ 9 months for almost half of the participants) may have been insufficient to demonstrate significant changes at an individual level and certainly at an organizational level. The project timelines also meant that there was a relatively brief period from commencement (and conclusion) of the intervention activities and post-intervention assessment. Dedicated embedding of the knowledge and skills gained within organizational structures was beyond the scope of this 3-year project. The attrition of participants from several organizations meant that a substantial proportion of participants engaged in limited intervention activities and furthermore were also unavailable for follow up surveying. The IRWFY instrument used to assess quantitative changes in research utilization lacked sensitivity to discern any intervention effect. The TROPIC program evaluation did not explore the barriers to evidence awareness, knowledge and uptake.

## Conclusions

In conclusion, the findings of this quantitative analysis of the TROPIC program indicated inconsistent and relatively modest effects at the individual level and no detectable effects at the organizational level. The findings are also inconsistent with previously reported qualitative results that indicate more positive effects arising from the same program. While design and measurement factors may partly account for the lack of quantitative effects, other contextual and program-specific factors may better explain the observed ineffectiveness and apparent inconsistency with previous qualitative findings. Future research is needed to better understand the barriers to implementation of future knowledge-broking interventions and the structural and organizational factors including those relating to evidence awareness, knowledge and uptake that may facilitate program effectiveness. Additionally, further work is needed to refine tools so they can more precisely quantify both individual and organizational level research utilization to enable better assessment of future knowledge broking programs.

## Abbreviations

IRWFY: “Is Research Working for You?”
NCDs: Non-communicable diseases
NGO: Nongovernmental organization
OPIC: Obesity Prevention in Communities
SPSS: Statistical Package for the Social Sciences
TROPIC: Translational Research for Obesity Prevention in Communities
WHO: World Health Organization
